# Enhanced early-life nutrition upregulates cholesterol biosynthetic gene expression and Sertoli cell maturation in testes of pre-pubertal Holstein bulls

**DOI:** 10.1038/s41598-019-42686-w

**Published:** 2019-04-23

**Authors:** Chinju Johnson, Alysha Dance, Igor Kovalchuk, John Kastelic, Jacob Thundathil

**Affiliations:** 1 Department of Production Animal Health, Faculty of Veterinary Medicine, University of Calgary, Calgary, AB T2N 4N1 Canada; 2 Department of Biological Sciences, University of Lethbridge, Lethbridge, AB TIK 3M4 Canada

**Keywords:** Developmental biology, Systems biology

## Abstract

Well-fed prepuberal Holstein bulls had larger testes, earlier puberty, higher LH, testosterone and IGF-1, earlier and more proliferating and differentiating Sertoli cells, and greater sperm production potential. The objective was to determine effects of pre-pubertal nutrition on mRNA expression of testicular genes. Holstein bull calves were fed high or low diets (20 or 12% crude protein, respectively and 71.6 or 64.4% Total Digestible Nutrients) from 2 wk, castrated at 8, 16, 24 and 32 wk and testicular mRNA extracted and sequenced. Differential expression of genes mainly occurred at 16 and 24 wk. At 16 wk, functional analysis (DAVID) of DE mRNA revealed common biological processes including “cholesterol” and “fatty acid biosynthesis,” with most genes (including HMGCR, HMGCS1, HSD17) upregulated in high-diet bulls (P < 0.05). Major pathways enriched at 16 wk were “cholesterol biosynthesis”, “steroid metabolism” and “activation of gene expression by Sterol regulatory element binding protein (SREBP)” (P < 0.05). In high-diet bulls, mature Sertoli cell marker Connexin 43, was upregulated at 16 wk and immature Sertoli cell marker (AMH) downregulated at 24 wk. There was an indirect interaction between insulin family receptor and most upregulated cholesterol biosynthesis genes. Pre-pubertal nutrition enhanced testicular cholesterol/steroid biosynthesis and Sertoli cell maturation.

## Introduction

It is well established that supplemental feeding of bulls early in life enhances reproductive development^1–3^. Pre-pubertal bull calves fed high nutrition (2–32 wk) had earlier puberty, larger testes and increased sperm production potential, without significant differences in sperm quality, compared to those on a low diet^4^. In addition, high-nutrition bulls had increased serum concentrations of LH, testosterone and IGF-I^5^. Earlier puberty and enhanced sperm production increase reproductive potential. However, cellular and molecular mechanisms underlying testicular changes in response to pre-pubertal nutrition remain largely unknown.

The pre-pubertal period (8–20 wk) in bulls is characterized by transient increases in peripheral gonadotropin concentrations and parallel increases in testosterone^1,6^. Serum LH concentrations start increasing during the late infantile period (4–5 wk), peak at ~12–16 wk and decline at ~25 wk^1,6^. In addition, serum FSH concentrations also increase during the pre-pubertal period in a non-pulsatile fashion, albeit not to the same extent as LH^6^. The magnitude of this early gonadotropin rise is crucial to initiate and support reproductive development in bulls^1^. Although testis growth is slow during the pre-pubertal period, it accelerates as puberty approaches, despite relatively low gonadotropin concentrations. Perhaps mechanisms triggered by early gonadotropin release or gonadotropin-independent mechanisms regulate testicular development in bulls.

There are major cytological and histological changes in bull testes during the pre-pubertal period. At ~8 wk, fetal Leydig cells degenerate and adult Leydig cells populate the intertubular component. Mesenchymal cells, comprising the rest of the compartment, cease proliferation at ~16 wk and transform to Leydig cells that proliferate until 30 wk, followed by decreased mitosis^7^. Steroidogenic enzymes and testosterone production are distinguishing features of mature Leydig cells^7^. Sertoli cells, the predominant tubular cell type during this period, proliferate from 4–8 wk, and after a decline, enter into a maturation phase (20 wk)^8^. Sertoli cell differentiation includes distinct morphological and functional changes to enable efficient spermatogenesis^9^. They undergo modifications in cell shape, nuclear location (basal), appearance of abundant smooth endoplasmic reticulum, ectoplasmic specializations, etc. The blood testes barrier, established by 24 wk, coincides with appearance of a tubular lumen. Mature Sertoli cells produce several peptides, including androgen binding protein, transferrin, cerruloplasmin and plasminogen activating factor. Furthermore, they promote germ cell proliferation by synthesis and secretion of pyruvate and lactate, the main energy source for germ cells^10^. There is also resumption of pro-spermatogonial proliferation, commencing between 4 and 16 wk and peaking at ~30 wk^8^, supported by increases in number of Sertoli cells and their metabolism. Finally, establishment of a functional blood testes barrier creates a tubular lumen, with emergence of primary spermatocytes and more advanced stages of spermatogenesis^8,11^.

Mechanisms by which better nutrition can enhance reproductive development was recently reviewed^12^. The long-standing assumption that there is a “metabolic sensor” in the brain capable of monitoring body metabolism and energy is at least a partial answer. It is assumed that this “sensor” analyzes blood concentrations of metabolic hormones, e.g. IGF-I and other nutrients and relays this information to the GnRH pulse generator in the hypothalamus^2,3^; this is required for GnRH neurons to over-ride negative feedback from testosterone and estradiol and thereby initiate pulsatile release of GnRH^1,2^. This promotes the anterior pituitary to release gonadotropins, which act on testes to promote reproductive development. Thus, enhanced pre-pubertal nutrition could hasten the early gonadotropin rise, puberty and sexual maturity in bulls^2^.

In the present study, bull calves were fed either a high or low diet from 2–32 wk of age, castrated at 8, 16, 24 or 32 wk and differential gene expression in testes determined. We hypothesized that an increase in IGF with a higher plane of nutrition (observed in our previous study^5^), is a major stimulus for reproductive development in pre-pubertal bulls.

## Results

### mRNA profile of testes

Approximately 480 million sequenced reads were obtained from the 28 libraries used in the present study. On average, 17,108,134 (SD = 3,353,629) reads were obtained per library and 15,699,023 (SD = 3,054,171) mapped to the Ensemble gene annotation database. Considering all age groups, 15,426,902 (SD = 3,388,812) reads from high-diet groups were mapped, compared to 15,903,113 (SD = 9,244,227) mapped from low-diet groups. On average, 91% of total reads were mapped to the bovine genome. A total of 19,994 genes were detected in the testicular tissue and their ensemble IDs are provided in Supplementary dataset 1. The most abundant gene in the testicular transcriptome of all libraries was cytochrome c oxidase subunit I (*COX1*), a mitochondrial gene (ENSBTAG00000043561), representing ~0.5% of total reads. Other abundant genes included cytochrome c oxidase subunit 3 (*COX3*, ENSBTAG00000043560), a mitochondrial gene and elongation factor 1-alpha 1 (*EEF1A1*, ENSBTAG00000014534), involved in RNA transport.

### Differentially expressed genes

At 8 wk, no genes were differentially expressed when comparing high- versus low-diet bulls. At 16 wk, 96 genes were differentially expressed (Supplementary dataset 2), of which 92 were upregulated in the high diet with genes *FDX1*, *HMGCR*, *HMGCS1*, *ACSS2* and *PRUNE2* expressing maximum log2 fold change. At 24 wk, a total of 87 genes were differentially expressed (Supplementary dataset 3), with 41 upregulated in high-diet bulls (genes with higher log2fold change- KRT8, ENPP3, CA3, HSD17B3). The list of downregulated genes at 24 wk included *AMH* and *DPT*, both negative regulators of cell proliferation. However, at 32 wk, only *ENPP3*, a nucleic acid binding gene was differentially expressed and was downregulated in the high-diet group. Accordingly, due to lack of differential expression at 8 and 32 wk, all further analyses were done only on 16- and 24-wk samples. In general, log fold changes varied from ±0.3 to ±2.56 among differentially expressed genes (DEGs). For visual representation of differential expression, MA plots were generated (log fold change and mean of normalised counts) and are available in Supplementary File. 1. In addition, scatterplots were generated to visualise transcriptomic differences across bulls from high- and low-diet groups and is provided in Supplementary File. 2.

#### GO term analysis

According to GO enrichment using DAVID, at 16 wk, most DEGs were associated with the biological process of “cholesterol biosynthesis”. Other significant GO terms included “isoprenoid biosynthesis”, “oxidation-reduction process” and “fatty acid biosynthesis” (Table 1). No GO terms were significantly enriched at 24 wk.

**Table 1:** GO (biological process) terms enriched using DAVID at 16 wk (adj P ≤ 0.05, count ≥2).

GO Term	Count	Adj P value
Cholesterol biosynthetic process	7	0.00
Isoprenoid biosynthetic process	5	0.00
Oxidation-reduction process	9	0.05
Fatty acid biosynthetic process	4	0.05

#### Enriched functions, pathways and upstream regulator analysis

**Figure 1:**
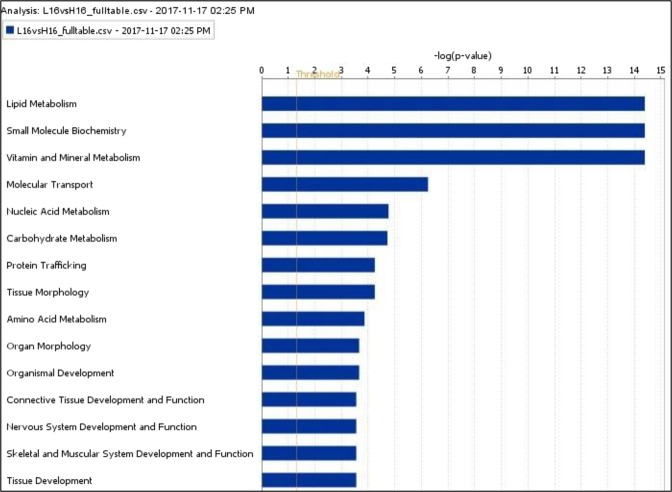
Top functions of genes differentially expressed at 16 wk as per IPA (top 15 functions shown). L and H stand for low and high diets respectively. Functions are marked on the X-axis and -log(p value) marked on the Y-axis. Right-tailed Fisher’s exact test was used to calculate the p-value determining the probability that each biological function assigned to that data set is due to chance alone (P < 0.05). The functions were generated through the use of “core analysis” in IPA (QIAGEN Inc., https://www.qiagenbioinformatics.com/products/ingenuity-pathway-analysis)^45^.

**Figure 2:**
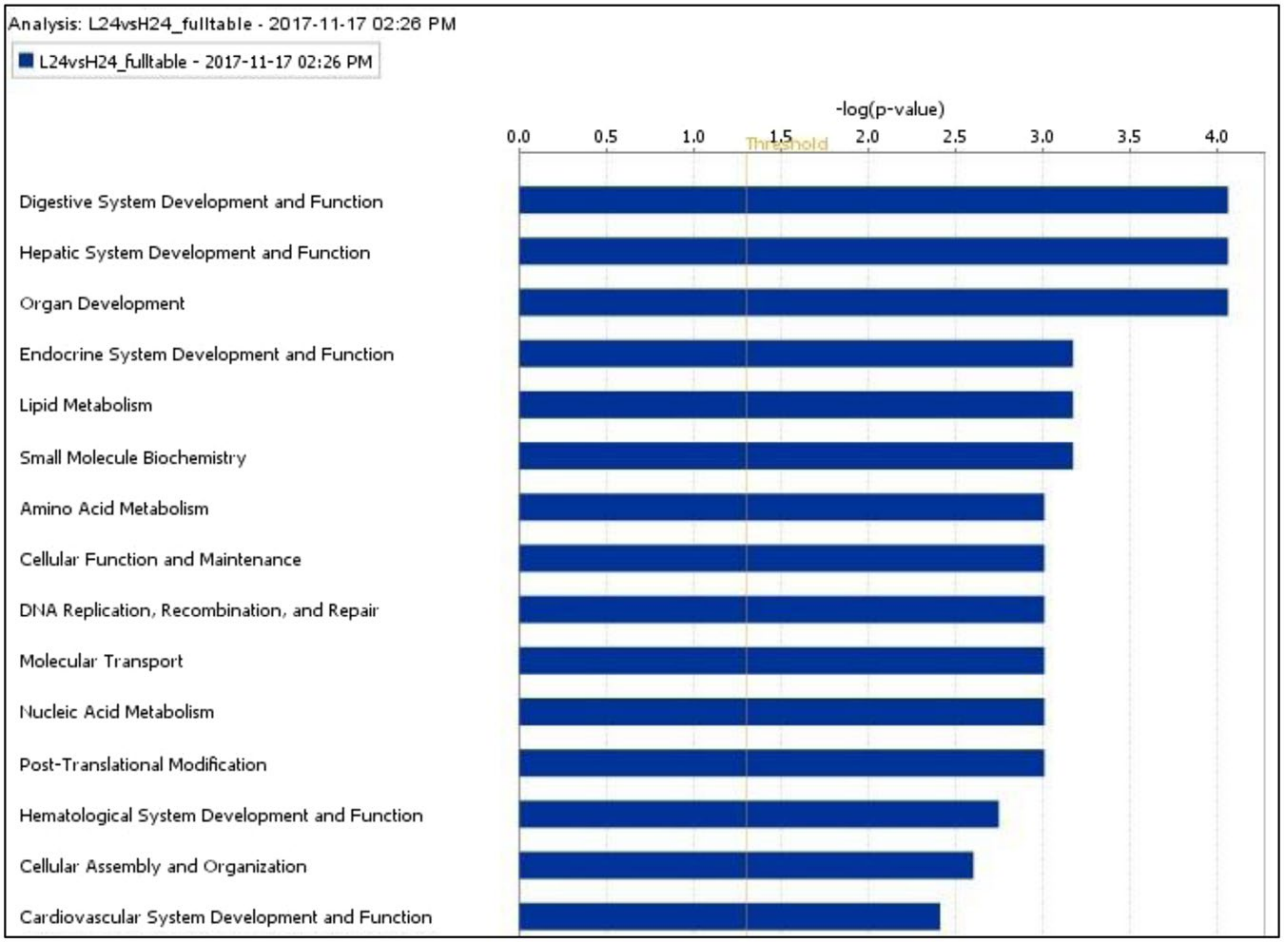
Top functions of genes differentially expressed at 24 wk as per IPA (Top 15 functions shown). L and H stand for low and high diets respectively. Functions are marked on the X-axis and -log(p value) marked on the Y-axis. Right-tailed Fisher’s exact test was used to calculate the p-value determining the probability that each biological function assigned to that data set is due to chance alone (P < 0.05). The functions were generated through the use of “core analysis” in IPA (QIAGEN Inc., https://www.qiagenbioinformatics.com/products/ingenuity-pathway-analysis)^45^.

Several functions were enriched at 16 wk, with major ones being “lipid metabolism”, “small molecule biochemistry” and “vitamin and mineral metabolism” (Fig. 1). Conversely, at 24 wk, “digestive system development and function”, “endocrine system development and function” and “lipid metabolism” were enriched (Fig. 2).

**Figure 3:**
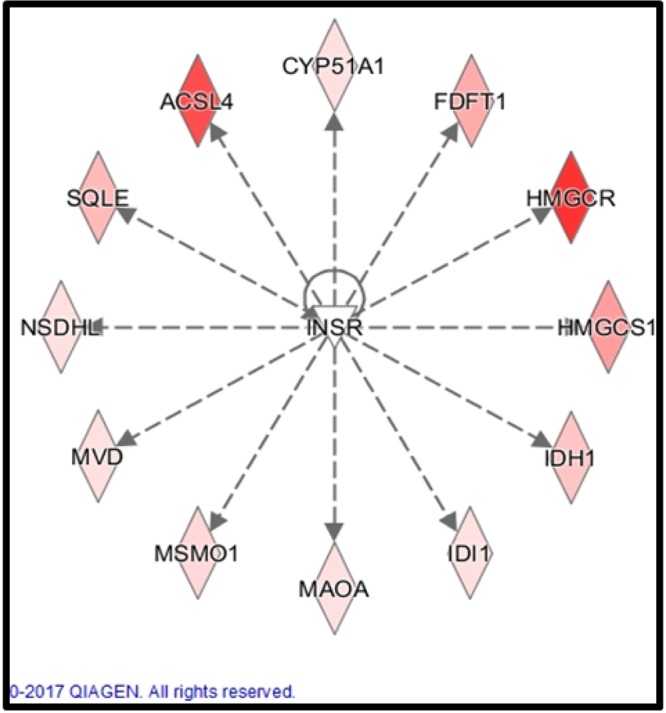
Associations of steroid biosynthesis genes (differentially expressed) to the insulin family receptor. Using IPA, network analysis overlaid with gene expression levels was generated on low vs high diet comparison made at 16 wk. Nodes represent genes and its colour indicates up (green) or down (red) regulation (low vs high diet); the brightness of color is related to the fold change of differentially expressed gene and the darker the color, the higher fold change. The dashed line indicates an indirect interaction between molecules as supported by information in the Ingenuity knowledge base. Generated through the use of “core analysis” in IPA (QIAGEN Inc., https://www.qiagenbioinformatics.com/products/ingenuity-pathway-analysis)^45^.

Major pathways enriched at 16 wk were “cholesterol biosynthesis”, “zymosterol biosynthesis”, “mevalonate pathways” (Supplementary File. 3). At 24 wk, “glutamine biosynthesis”, and “creatinine biosynthesis” were enriched (Supplementary File. 4). Major upstream regulators at 16 wk included SCAP, SREBP1, SREBP2, PPARG and testosterone were predicted to undergo activation in high-diet bulls (z score ≥ 2, P ≤ 0.05, Supplementary dataset 4). INSIG1, INSIG2, PTEN and LY294002 (latter two- negative regulators of PI3K signaling) were inhibited in high-diet bulls. Network analysis using IPA also revealed an indirect interaction of steroid biosynthetic genes to the insulin family receptor (Fig. 3).

#### Pathway analysis

Pathway analysis with REACTOME browser revealed enriched pathways including “cholesterol biosynthesis”, “steroid metabolism”, “activation of gene expression by Sterol regulatory element-binding protein (SREBP)” and “fatty acyl-CoA biosynthesis” at 16 wk (Table 2).

**Table 2:** REACTOME pathway analysis for DEGs at 16 wk. (FDR < 0.05, count ≥ 2).

Pathway name	Count	FDR value
Cholesterol biosynthesis	11	0.000
Metabolism	43	0.000
Metabolism of steroids	14	0.000
Activation of gene expression by SREBF (SREBP)	8	0.000
Metabolism of lipids	24	0.000
Regulation of cholesterol biosynthesis by SREBP (SREBF)	8	0.000
Fatty acyl-CoA biosynthesis	5	0.000
Synthesis of very long-chain fatty acyl-CoAs	3	0.042
Metabolism of vitamins and cofactors	7	0.045

### RT-qPCR validation of differentially expressed genes

**Figure 4:**
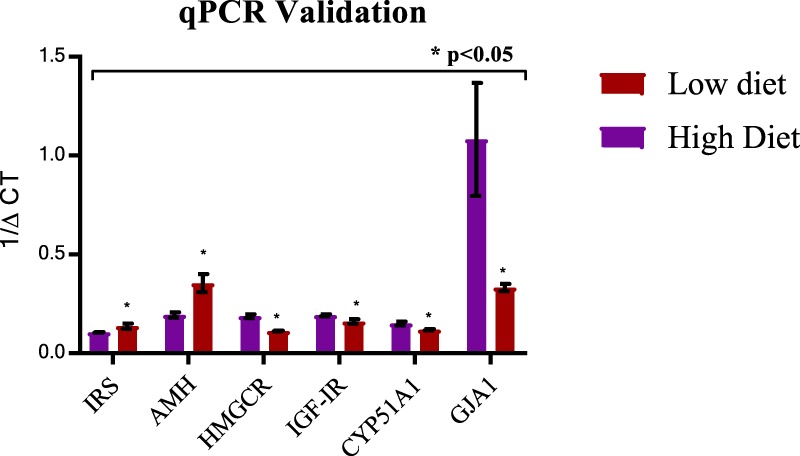
qPCR validation of differentially expressed genes IRS (24wk), AMH (24 wk), HMGCR (16wk), CYP51A1 (16wk), GJA1 (16wk) and IGF-IR (16 wk) in Holstein bull calves fed a low or high diet. Data are presented as Mean ± SEM. *P < **0**.**05**.

A cohort of five mRNAs from the DE mRNAs and *IGF-IR* (p < 0.1) were selected for validation; RT-qPCR results were consistent with sequencing data for all of them (Fig. 4). The PCR primer sequences were designed using National Center for Biotechnology and Information (NCBI) Primer blast^13^ and purchased from Thermo Fisher Scientific (Table 3).

**Table 3:** Primer sequences used for validation of differentially expressed genes in the testicular tissue of Holstein bull calves.

Gene	Abbreviation	PCR Primer sequence	Product size (bp)	NCBI Accession No
Glyceraldehyde-3-phosphate dehydrogenase	GAPDH	F: GCATCGTGGAGGGACTTATGA	67	NM_001034034.2
		R: GGGCCATCCACAGTCTTCTG		
Anti-mullerian hormone	AMH	F: GTGACTTGACCACCTTCGCA	160	NM_173890.1
		R: AACCTCAGCAAGGGTGTTGG		
3-Hydroxy-3-Methylglutaryl-CoA Reductase	HMGCR	F: AATCTGGCAGCTCAGCCATT	114	NM_001105613.1
		R: TTGCCAGAGGGAAACACTCG		
Insulin receptor substrate 4	IRS4	F: CCTTTTCGGAGCTCCCCATT	184	XM_005227898.3
		R: TGGAAGGTGATCCCCCATCT		
Insulin-like growth factor 1 receptor	IGF-1R	F: TCAAGAGTTATCTCCGGTCTCTGA	91	NM_001244612.1
		R: CCAGCCATCTGGATCATTTTG		
Gap junction protein alpha 1	GJA1	F: TACAGCCTCTCGCCATTGTG	193	NM_174068.2
		R: GGCTGTTGAGTACCACCTCC		

## Discussion

To determine changes in the mRNA expression of genes associated with differential pre-pubertal nutrition, bull calves were fed either a high or low diet from 2 to 32 wk, with serial castrations done at 8, 16, 24 and 32 wk. To our knowledge, this was the first study using a genome-wide analysis of testicular tissue obtained at various time points, i.e. through the entire pre-pubertal period in Holstein bulls. In a previous phenotypic study, we identified enhanced gonadotropins (LH), testosterone, IGF-I secretion, early puberty and better sperm production in bulls fed a high vs low diet during their pre-pubertal period^4,5^. Differences in gene expression within testes were identified at 16 wk and perhaps could have been detected earlier with more frequent sampling. According to our main findings, higher *IGF-IR* expression and elevated IGF-I concentrations^5^ promoted cholesterol biosynthesis and Sertoli cell maturation to hasten puberty and sperm production potential in pre-pubertal bulls.

As mentioned previously, testes undergo numerous changes prior to puberty, with the least changes occurring during the infantile period^1^. From 2–8 wk, bull calves were fed high (8 L) or low (4 L) volumes of milk replacer, depending on the experimental group. Genome-wide analysis of differential gene expression revealed lack of differential expression between groups at 8 wk (P < 0.05). Since nutritional modulation was not restricted to infantile or pre-pubertal periods in our study, it was not possible to completely disregard the importance of high infantile nutrition. However, restricted feeding in Holstein bull calves in the first 3 wk of life reduced average daily gain (ADG) and resulted in bulls that were lighter and had lower testosterone concentrations at 10 wk, than calves fed *ad libitum*, suggesting a positive effect^14^.

The early gonadotropin rise is a marked feature of the pre-pubertal period. Increases in LH and FSH are due to pulsatile releases of GnRH. Cholesterol biosynthetic genes (including *HMGCR*, *HMGCS1*, *ACAT2* and *SQLE*) and genes involved in steroid metabolism (including *HSD17B7*, *HSD17B7* and *CYP51A1*) had higher expression in high-diet bulls at 16 wk, suggestive of an early rise in steroid hormone synthesis compared to low-diet bulls. Biosynthesis of cholesterol, the precursor of steroid hormones, is mainly regulated by a membrane bound transcription factor called SREBP^15^. When cleaved by SREBP cleavage activating protein (SCAP), the inactive transcription factor is rendered active and it promotes activation of enzymes and receptors facilitating cholesterol synthesis and uptake. Among the many upstream regulators predicted to be activated in high-diet bulls were SCAP, SREBP and PPARG. The latter, also known as peroxisome proliferator-activated receptor gamma, is a ligand-dependant nuclear receptor involved in lipid metabolism and cholesterol homeostasis^16^.

Other marked changes during this interval involve proliferation and differentiation of Sertoli cells. Connexin-43 (*GJA1*) a Sertoli cell gap junctional protein and maturation marker^17^, had higher expression in high-diet bulls. In addition, *AMH*, a marker of immature Sertoli cells^9^ was downregulated in high-diet bulls at 24 wk. During pre-puberty, *AMH* gene expression starts reducing with enhanced androgen receptor (*AR*) expression with increased FSH and testosterone secretion^18,19^. Maintaining spermatogenesis by providing nutritional and energy support is a predominant function of Sertoli cells. Lactate, the major energy source for germ cells, is synthesized from glucose in Sertoli cells^20^. In addition to glucose, free fatty acids, apoptotic germ cells, residual bodies and amino acids are additional sources for lactate production in Sertoli cells. Through the less efficient glycolytic process, pyruvate synthesized from glucose is mainly converted to lactate and to a lesser extent, to amino acids. In germ cells, lactate is converted to pyruvate and used for acetyl CoA synthesis by pyruvate dehydrogenases (PDH) and is further used by mitochondria^20^.

Cholesterol biosynthesis appeared to be an important factor altered by differential diets in pre-pubertal bulls. Cholesterol has important roles in both steroidogenesis and spermatogenesis. Cholesterol, a 28-carbon steroid, is the precursor of all steroid hormones, and therefore regulates steroid hormone production in Leydig cells. It is also a main energy source for Sertoli cells and with its role in sperm membrane remodeling, cholesterol is a crucial regulator of spermatogenesis. Accordingly, cholesterol is important for germ cell maturation and a pre-requisite for mass production of sperm. During spermatogenesis, sperm membranes are loaded with cholesterol, synthesised by germ^21^ or Sertoli cells^22^ or via influx from circulation with cholesterol transporters^23^. However, during epididymal transport, an efflux of cholesterol occurs, increasing membrane fluidity, and promoting sperm maturation^24^. A positive correlation between membrane cholesterol content and sperm quality has been reported^25^. Sertoli cells also acquire cholesterol from apoptotic germ cells and residual bodies which are esterified and stored as lipid droplets^26^. In addition, to maintain homoeostasis, excess cholesterol is returned to circulation by HDL-based reverse cholesterol transport^27^.

Thus, better pre-pubertal nutrition augmented cholesterol/steroid biosynthesis and Sertoli cell maturation, thereby promoting spermatogenesis. In the phenotypic study, IGF-I concentrations were high in high-diet bulls from 11–32 wk, i.e., through most of the differential feeding period, raising a question of whether IGF signaling is partly responsible for observed changes. The essential role of IGF-I in Sertoli cell proliferation and maturation have been reported^28,29^. Accordingly, deletion of *IGF-IR* and *INSR* in mice, lowered Sertoli cell proliferation, resulting in 70% reduction in testes size. In addition to IGF-I, FSH is a major endocrine hormone associated with Sertoli cell proliferation; however, serum FSH concentrations were neither increased in the animal study nor in our present gene expression study. Furthermore, FSH requires IGF signaling to mediate effects on immature Sertoli cells^28^, supporting our idea of IGF being the major intratesticular signal. IGF receptors are also present on Leydig cells; IGF-I promotes Leydig cell proliferation and exogenous IGF-I increased LH secretion in sheep^30^.

Numerous genes involved in steroid biosynthesis indirectly interacted with the Insulin family receptor (*INSR*), suggestive of their involvement in the same (Fig. 3). Within the Insulin like growth factor family, both *INSR* and *IGF-IR* share 84% similarity in the β subunit and 64–67 and 100% similarly in the α subunit and ATP binding domain, respectively^31,32^. Furthermore, IGF-I ligand with a high affinity to its cognate receptor (IGF-IR), can also bind to the Insulin receptor with low affinity. Based on our earlier phenotypic study, increased serum IGF-I concentrations (and lack of differences in serum insulin concentrations) suggest critical involvement of IGF-I and its receptor when compared to the INSR. Accordingly, in mice liver, partial deficiency of IGF-I reduced expression of *HMGCR* and *HMGCS1* involved in cholesterol biosynthesis^33^. In a similar manner, IGF enhanced expression of genes involved in cholesterol and fatty acid biosynthesis in murine myoblasts^34^. Furthermore, cholesterol biosynthesis was lowered with inhibition of IGF-I mediated PI3K/Akt signaling^35^. In addition, IGF-I also enhanced transport of SCAP, facilitating the escort of SREBP from endoplasmic reticulum to Golgi complex, which was further blocked by a PI3K inhibitor^35^; this supported our concept of a direct impact of IGF on steroid biosynthesis in bovine testes. However further studies are needed to confirm this interaction.

**Figure 5:**
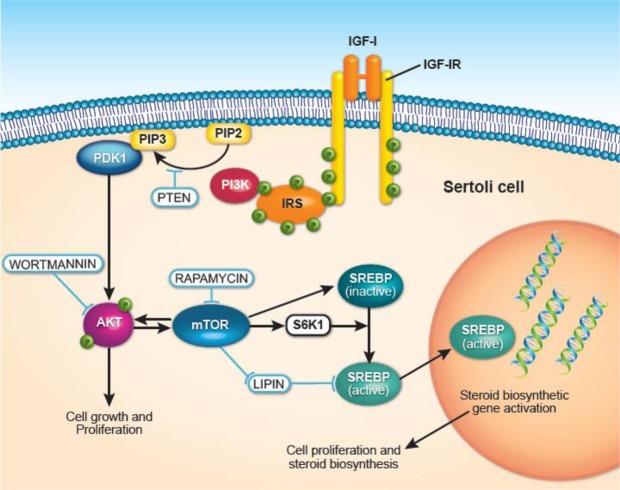
Hypothetical model for IGF-I signaling and steroid biosynthesis in bovine Sertoli cells. IGF-I signaling via PI3K/Akt/mTOR signaling in Sertoli cells. In response to binding with the ligand IGF-I, IGF-IR undergoes tyrosine phosphorylation, followed by the activation of insulin receptor substrates and in turn PI3K. Phosphatidyl inositol diphosphate (PIP2) is converted to Phosphatidyl inositol triphosphate (PIP3) which activates PDK1. This process is inhibited in the presence of PTEN, a potent inhibitor for conversion of PIP2 to PIP3. Akt phosphorylation caused by PDK1 can mediate cell growth and proliferation or activate downstream mTOR signaling. Wortmannin and Rapamycin are potent inhibitors of Akt and mTOR phosphorylation, respectively. Phosphorylated mTOR or S6K1 can facilitate activation of SREBP from its inactive form in the endoplasmic reticulum to an active form which enters the nucleus. Active SREBP, a transcription factor, facilitates activation of genes associated with steroid biosynthesis and promotes cell proliferation.

In conclusion, enhanced pre-pubertal nutrition in Holstein bulls enhanced testicular cholesterol/steroid biosynthesis and Sertoli cell maturation to promote early reproductive development. A hypothetical model for IGF-I signaling in Sertoli cells has been provided in Fig. 5. IGF-I is a major factor regulating Sertoli cell proliferation and maturation in bulls. In addition, Insulin family receptor was indirectly associated with most cholesterol biosynthesis genes through SREBP/SCAP signalling.

## Materials and Methods

### Ethics approval

This experiment was conducted in accordance with guidelines of the Canadian Council on Animal Care and was reviewed and approved by the University of Calgary Veterinary Sciences Animal Care Committee.

### Calves and treatments

Twenty-eight bull calves (1 wk old) were randomly allocated into two nutritional groups and provided either a high or low diet from 2 to 32 wk of age. From 2 to 8 wk of age, calves were fed reconstituted milk replacer at the rate of 8 or 4 L/d for high or low diets, respectively. Calves were then transitioned onto a barley silage-based diet (forage source for all diets) and fed a high- or low-nutrition diet from 8 to 32 wk (Table 4). The high-nutrition diet had 20% CP and 71.6% TDN (dry matter basis), whereas the low-nutrition diet had 12% CP and 64.4% TDN. These diets were based on those in a previous study^5^, with feed intake controlled as described^5^.

**Table 4:** Composition of low- and high-nutrition diets (as fed %) for Holstein bull calves from 8 to 32 wk of age.

Ingredient	Low	High
Barley silage	98.5	55.5
Rolled barley	—	26.0
Canola meal	—	15.9
Minerals & vitamins	1.5	2.6

### Castrations

Bull calves from each nutrition group were surgically castrated at 8 (n = 6, high-2, low-4), 16 (n = 7, high-3, low-4), 24 (n = 8, high-4, low-4), or 32 wk (n = 7, high-3, low-4) of age. Castrations were done under caudal epidural anesthesia (0.07 mg/kg BW of xylazine; Rompun, Bayer Inc., Mississauga, ON, Canada; in 3 mL saline) and calves were given 0.5 mg/kg meloxicam (Metacam 20; Boehringer Ingelheim, Burlington, ON, Canada). Testes were recovered and weighed. Testicular parenchyma was cut into cubes (~10 mm in each dimension) and snap-frozen on dry ice.

### RNA Isolation

A small portion of testicular parenchyma (~75 mg) from each bull (n = 28) was homogenised and total RNA extracted. Trizol reagent-mediated extraction was performed as per manufacturer’s instructions (Invitrogen, Canada, Burlington, ON, Canada). Total RNA was re-suspended in water, quality and quantity measured using a Nanodrop via spectrophotometry (2000/2000c, Thermo Fisher Scientific, Waltham, MA, USA). RNA integrity number (RIN) was assessed on an Agilent 2100 bioanalyzer using the Agilent RNA 6000 Nano kit (Agilent Technologies, Santa Clara, CA, USA).

### RNA sequencing of testicular tissue

#### Library preparation and sequencing

For each sample (n = 28) total RNA (1 µg) was used to create mRNA libraries using the TruSeq Stranded mRNA Library Prep Kit (Illumina, San Diego, CA, USA) at the Plantbiosis Ltd (Lethbridge, AB, Canada) sequencing facility. After library quantification, individual libraries were diluted to a final concentration of 4 nM, pooled (n = 28) and sequenced using the NextSeq. 500 system (Illumina). Sequencing was performed as 75 base pair single-end reads. Base calling and demultiplexing were done with CASAVA 1.9 (Illumina) with default settings. Initial quality control, conducted with FastQC detected no adapter sequences, base qualities and other parameters were excellent.

#### Mapping and annotation of reads

Sequenced reads were aligned to the bovine genome (*Bos taurus*, UMD3.1.87, downloaded from Illumina iGENOME website) using TopHat^36^, with Bowtie2^37^ as an internal aligner. FeatureCounts program was used to count number of reads on each exon by transcript meta-feature^38^. To conduct a “per-gene” analysis, the most expressed transcript over all conditions was selected by the provided *ad hoc* python script (*select_protein_coding_transcripts*.*py*). This script enables extraction of all transcripts corresponding to protein coding genes from the EnsemblUMD3.1.87 annotation file (GFF). Then, all count-tables obtained with FeatureCounts was loaded, and the count in each sample normalised by library size (mapped reads in the sequencing library). If the gene had more than one transcript, the transcript with the highest number of reads assigned was used in the analysis.

#### Detection of differentially expressed genes

Normalised counts on protein coding genes were used for analysis of differential expression with DESeq. 2^39^ in R package (Version 1.14.1) via SARTools6 (Version 1.4.0) package^40^. DESeq. 2 package has a built-in independent filtering procedure enabled and is described here https://www.bioconductor.org/packages/devel/bioc/vignettes/DESeq.2/inst/doc/DESeq.2.html#indfilt. So, pre-filtering for lowly expressed genes was not performed. Genes differentially expressed between experimental conditions were detected using the Wald test on normalised count data. Benjamini-Hochberg multiple comparisons correction procedure^41^ was done to determine an adjusted P value (genes P < 0.05 were considered differentially expressed).

### Candidate gene- qPCR validation of differentially expressed genes

Real-time reverse transcription polymerase chain reaction (RT-PCR) was performed as described^42^. Reverse transcription followed by PCR with SYBR Green (Fast SYBR® Green Master Mix; Applied Biosystems) was used to validate mRNA expression of five differentially expressed genes: *GJA1* (16 wk), *CYP51A1* (16 wk), *AMH* (24 wk), *HMGCR* (16 wk), *IRS* (24 wk) and *IGF-IR* (16 wk, included due to differential levels in the blood of bulls based on our phenotype study). The PCR primer sequences were designed using National Center for Biotechnology and Information (NCBI) Primer blast (Table 3) and obtained from Thermo Fisher Scientific. Total RNA (1 µg) was treated with RNase-free DNase I (Thermo Fisher Scientific) for 15 min at RT, followed by addition of 25 mM EDTA and heat denaturation at 65 °C for 10 min. Reverse transcription was performed using High Capacity cDNA Reverse Transcription Kit and random hexamers (Applied Biosystems). A 1:10 dilution of cDNA was efficient with all primers and was used for PCR amplification. All PCR were performed in 96-well plates (Applied Biosystems) and cycling conditions were as follows: 95 °C for 10 min, 40 cycles of 95 °C for 15 s and 59 °C for 1 min, and 55–95 °C (dissociation). Every PCR well reaction contained: Fast SYBR green Master mix- 7.5 μl, forward primer (20 pmol/μl)−0.5 μl, reverse primer (20 pmol/μl)−0.5 μl, cDNA template-0.5 μl and water-1 μl. All reactions were duplicated with inclusion of a non-RT and no-cDNA controls. Melt curve analysis was performed to evaluate product purity. Cq (mean threshold cycle) values obtained were normalised against a reference gene glyceraldehyde-3-phosphate dehydrogenase (*GAPDH*)^42^. The ∆Cq values so obtained were expressed as 1/∆Cq to facilitate visual interpretation of gene expression. Independent Student’s *t*-tests were performed to detect significant differences in gene expression (P < 0.05). R software (v 3.4.3) was used to perform all statistical analyses^43^.

### Functional analysis of DE mRNA

Ensembl identities of DEGs from 16 and 24 wk were uploaded to Database for Annotation, Visualization and Integrated Discovery (DAVID) to detect enriched Gene Ontology Biological processes (GO)^44^. The functional annotation and clustering option was used for most analyses. All significant GO terms with an adjusted P ≤ 0.05 and molecule number ≥2 were reported. All 19,994 Ensembl IDs expressed in our study were included as background for analysis in DAVID (Supplementary dataset 1).

Ingenuity pathway analysis (IPA; QIAGEN Inc., https://www.qiagenbioinformatics.com/products/ingenuitypathway-analysis) was used to detect the top most functions, pathways and upstream regulators of differentially expressed genes^45^. The entire dataset containing differentially expressed Ensembl Gene IDs, log fold change, and P-values for comparison between high and low diets was uploaded into IPA (v7.0). Ingenuity pathway knowledge database (genes) was used as the reference dataset for all analysis in IPA. For functional analysis, Right-tailed Fisher’s exact test was used to calculate the p-value determining the probability that each biological function assigned to that data set was due to chance alone. For pathway analysis, significant differences were measured based on both ratio (number of molecules from the data set that map to the pathway divided by the total number of molecules that map to the canonical pathway) and Fisher’s exact test-based p-value. Upstream regulator analysis predicts the cascade of upstream transcriptional regulators to explain the differential gene expression dataset. This analysis uses Ingenuity Knowledge base with stored information on prior expected effects between transcriptional regulators and their target genes. In addition, this analysis computes an activation z-score predicting status of the upstream regulator (activated or inhibited). Activation or inhibition was declared for z-score ≥±2.

For pathway analysis, DEGs were uploaded to REACTOME pathway browser and pathways were declared significant at false discovery rate (FDR) < 0.05 and molecule number ≥2^46,47^.

## Supplementary information

Supplementary files 1, 2, 3 and 4

Dataset 1

Dataset 2

Dataset 3

Dataset 4

## Data Availability

All data generated or analysed during this study were deposited in the publicly available NCBIs Gene Expression Omnibus Database. Datasets are accessible through GEO Series accession number GSE125082 (https://www.ncbi.nlm.nih.gov/geo/query/acc.cgi?acc=GSE125082).
