# Supplementary material for: Enhanced early-life nutrition upregulates cholesterol biosynthetic gene expression and Sertoli cell maturation in testes of pre-pubertal Holstein bulls
Source: Sci Rep. 2019 Apr 23;9:6448. doi: 10.1038/s41598-019-42686-w (PMC6478835; doi:10.1038/s41598-019-42686-w)
Supplement: Supplementary file 1 — Supplementary files 1, 2, 3 and 4 [file 41598_2019_42686_MOESM1_ESM.pdf]

Enhanced early-life nutrition upregulates cholesterol biosynthetic gene expression and Sertoli cell maturation in testes of pre-pubertal Holstein bulls

Chinju Johnson[1], Alysha Dance[1], Igor Kovalchuk[2], John Kastelic[1] and Jacob Thundathil[1*]

[1]Department of Production Animal Health, Faculty of Veterinary Medicine,

University of Calgary, Calgary, AB T2N 4N1, Canada

[2]Department of Biological Sciences, University of Lethbridge,

Lethbridge, AB, TIK 3M4, Canada

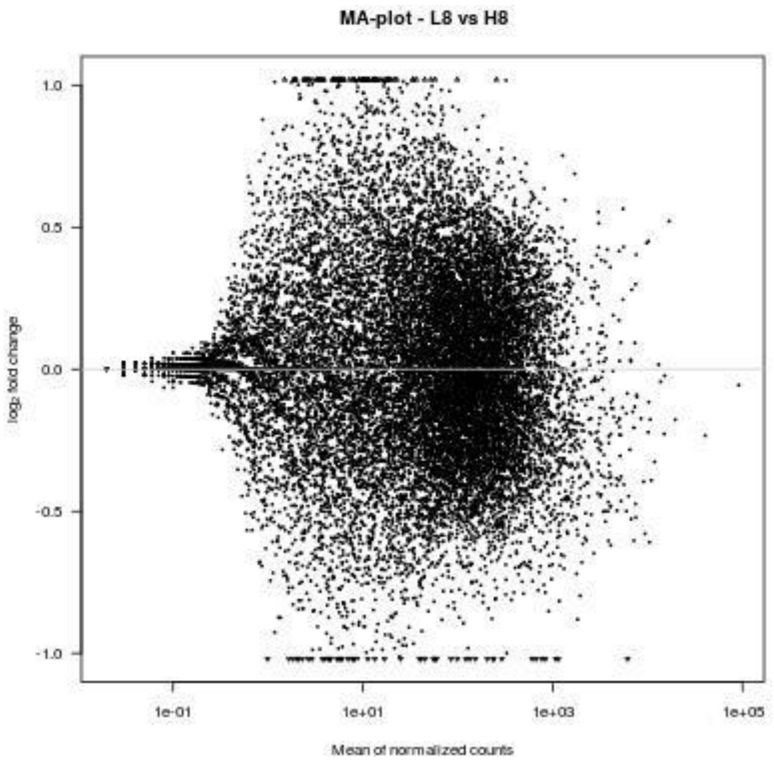
MA-plot - L8 vs H8
1.0
0.5
0.0
-0.5
-1.0
log2 fold change
1e-01
1e+01
1e+03
1e+05
Mean of normalized counts

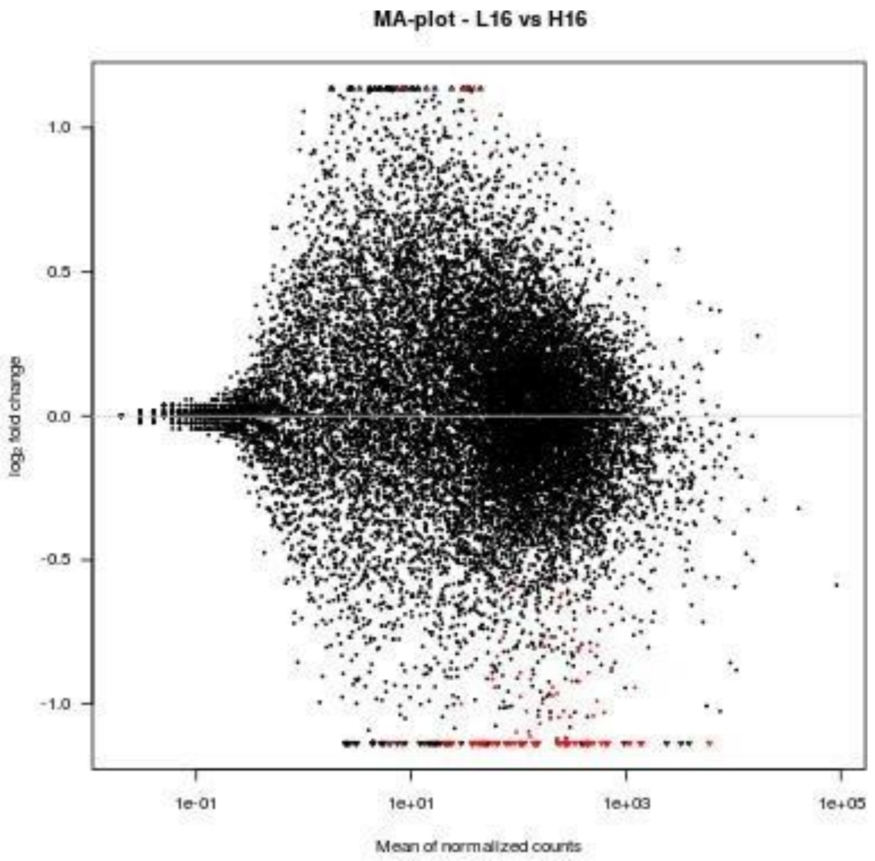

MA-plot - L16 vs H16
1.0
0.5
0.0
-0.5
-1.0
log2 fold change
1e-01
1e+01
1e+03
1e+05
Mean of normalized counts

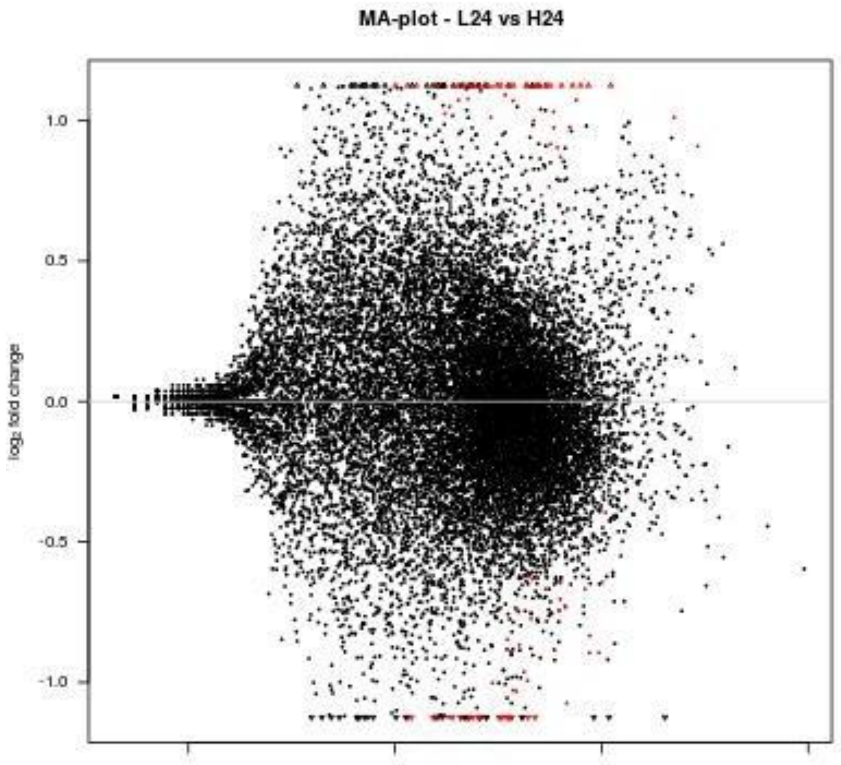

MA-plot - L24 vs H24
1.0
0.5
0.0
-0.5
-1.0
log2 fold change

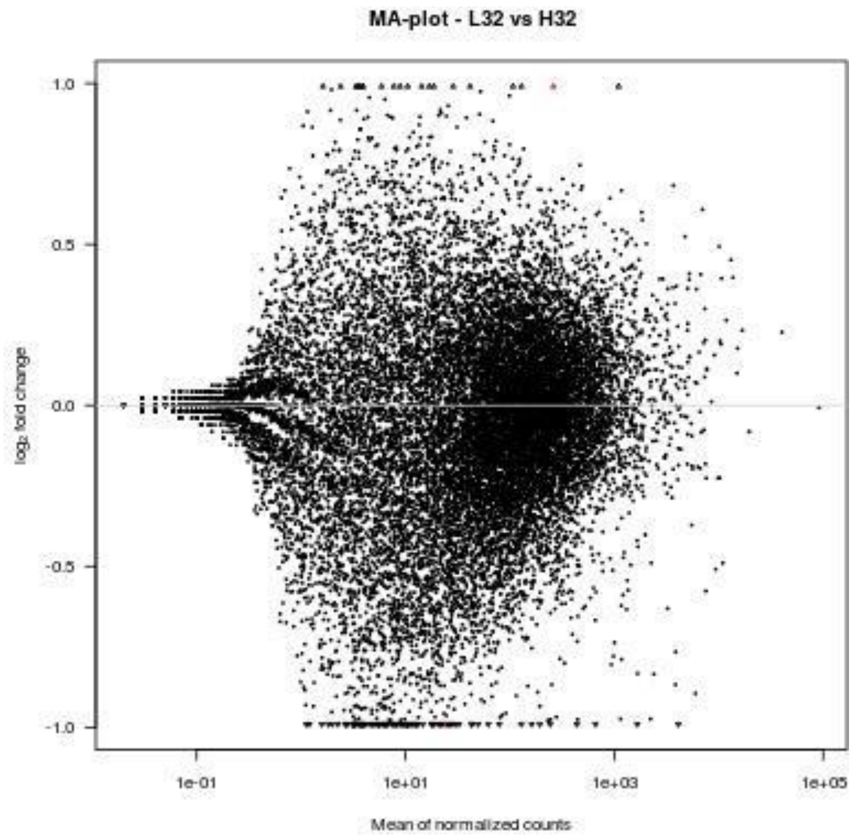

Supplementary File 1. MA plots (visual interpretation of gene expression data) for genes expressed in the low- vs high- diet comparison made at 8, 16, 24 and 32 wk. L and H represent low and high diets respectively. Differentially expressed genes ($p<0.05$) are highlighted in red, others in black. X axis- Mean of normalized counts, Y axis- log2 fold change.

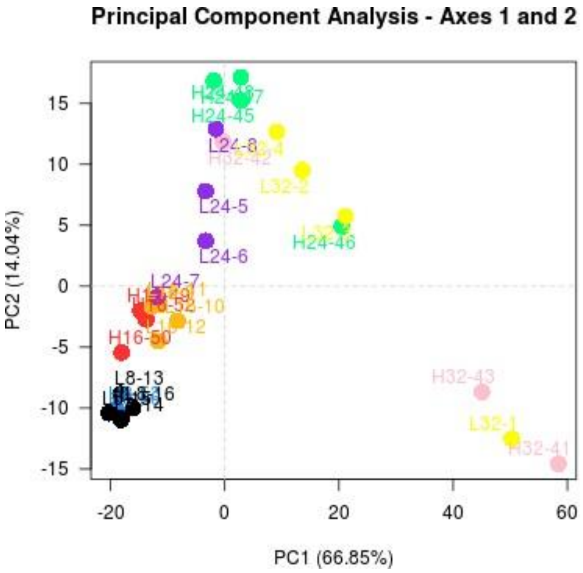
Principal Component Analysis - Axes 1 and 2
PC1 (66.85%)
PC2 (14.04%)
-20
0
20
40
60
15
10
5
0
-5
-10
-15
H24-45
L24-8
L24-5
L32-2
H24-46
L24-6
L24-7
H16-50
L8-13
H32-43
L32-1
H32-41

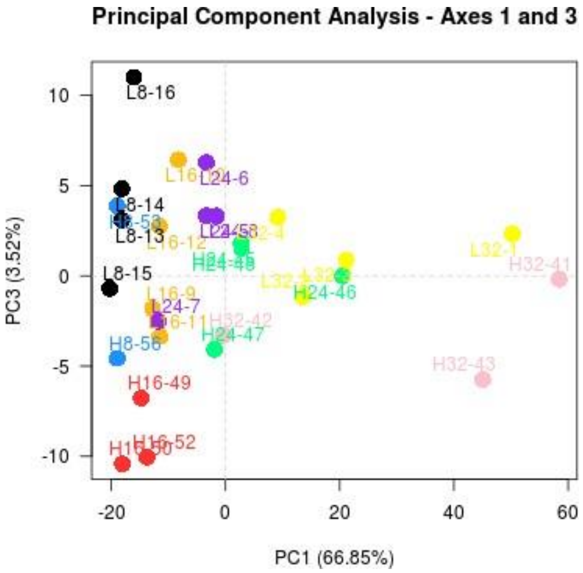
Principal Component Analysis - Axes 1 and 3
PC1 (66.85%)
PC3 (3.52%)
-20
0
20
40
60
10
5
0
-5
-10
L8-16
L24-6
L8-14
L8-13
L16-12
L8-15
H24-46
L16-9
L24-7
H32-42
H24-47
L32-1
H32-41
H8-56
H32-43
H16-49
H16-52

Supplementary File 2. Scatterplot with transcriptomic differences across bulls from low and high-diet groups using principal component analysis (PCA) (Axes used: PC1 66.85%, PC2 14.04%, PC3 3.52%). L and H stands for low and high diets respectively. The number immediately following L or H represent the week (8, 16, 24 or 32) they were castrated. The second number indicates the bull ID.

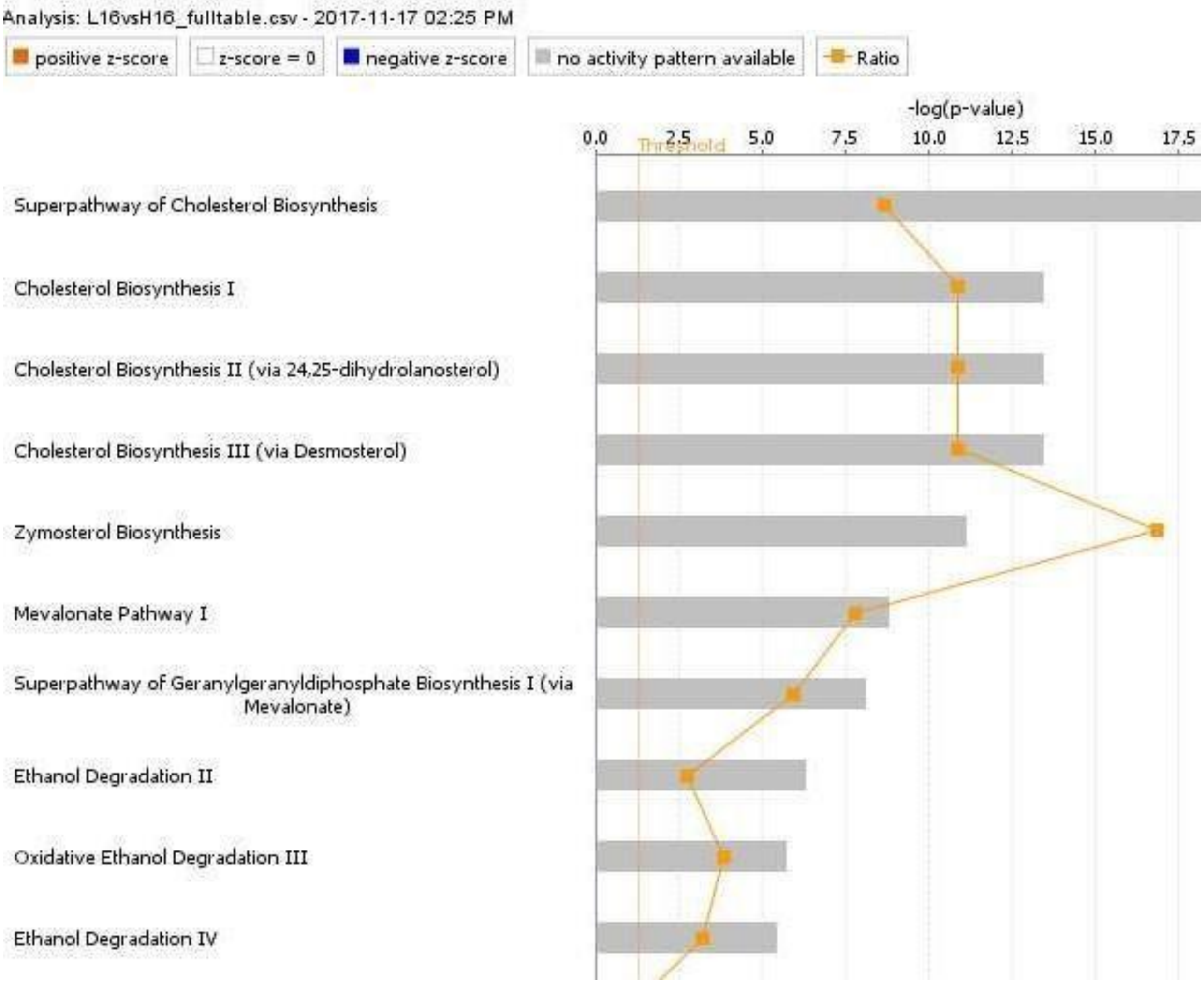

Analysis: L16vsH16_fulltable.csv - 2017-11-17 02:25 PM
positive z-score
z-score = 0
negative z-score
no activity pattern available
Ratio
-log(p-value)
0.0
2.5
5.0
7.5
10.0
12.5
15.0
17.5
Threshold
Superpathway of Cholesterol Biosynthesis
Cholesterol Biosynthesis I
Cholesterol Biosynthesis II (via 24,25-dihydrolanosterol)
Cholesterol Biosynthesis III (via Desmosterol)
Zymosterol Biosynthesis
Mevalonate Pathway I
Superpathway of Geranylgeranyldiphosphate Biosynthesis I (via Mevalonate)
Ethanol Degradation II
Oxidative Ethanol Degradation III
Ethanol Degradation IV

Supplementary File 3. IPA analysis for pathways enriched at 16 wks (Top 10 pathways shown). L and H represent low and high diet respectively. The colour of each pathway (bar) indicates its direction of change; blue (positive z score)- up-regulated, orange (negative z score)- down-regulated, grey- no activity pattern available or white z score=0 (white). Different pathways are marked on the X-axis and -log(p value) marked on the Y-axis. Significant differences were measured in 2 ways: 1) Ratio (orange squares)- number of molecules from the data set that map to the pathway divided by the total number of molecules that map to the canonical pathway is displayed. 2) Fisher's exact test was used to calculate a p-value determining the probability that the association between the genes in the dataset and the canonical pathway is explained by chance alone (P< 0.05). The pathways were generated through the use of "core analysis" in IPA (QIAGEN Inc., https://www.qiagenbioinformatics.com/products/ingenuity-pathway-analysis) [1].

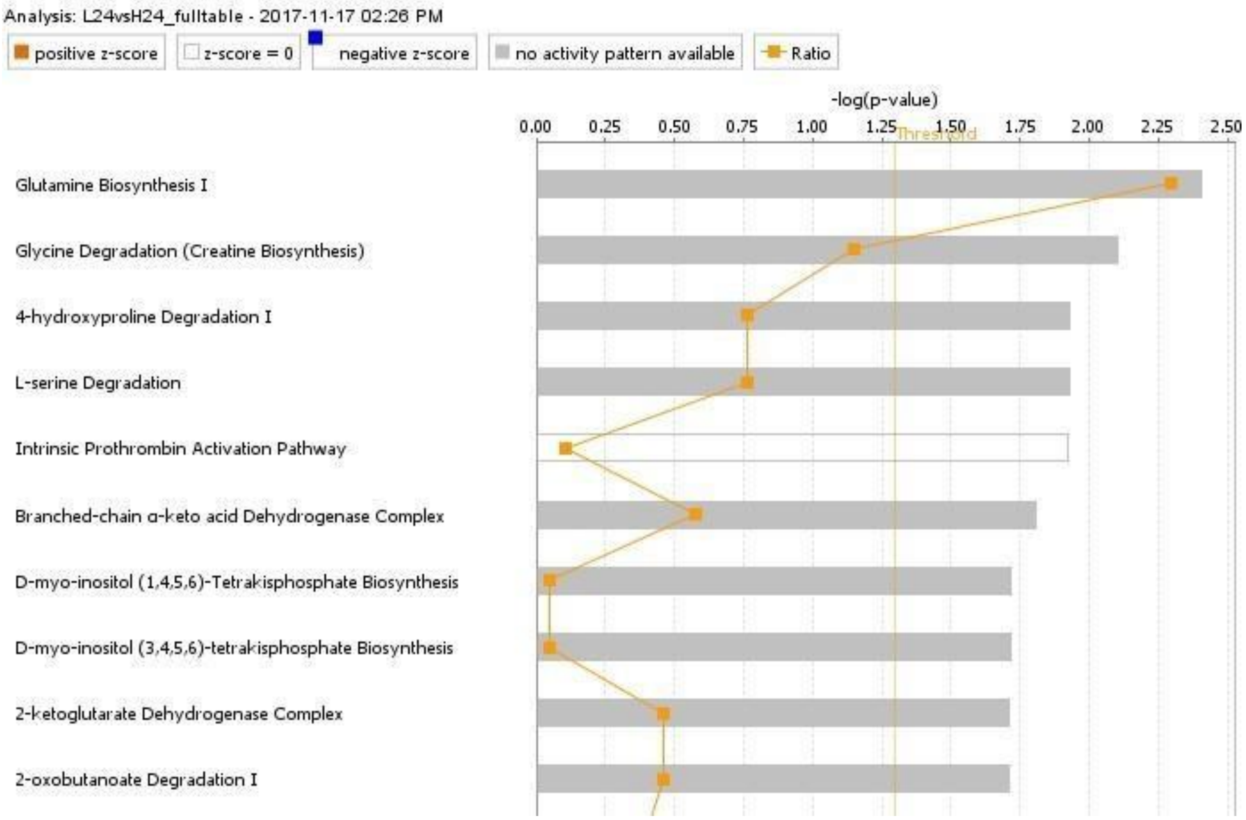

Analysis: L24vsH24_fulltable - 2017-11-17 02:26 PM
positive z-score
z-score = 0
negative z-score
no activity pattern available
Ratio
-log(p-value)
0.00
0.25
0.50
0.75
1.00
1.25
1.50
1.75
2.00
2.25
2.50
Threshold
Glutamine Biosynthesis I
Glycine Degradation (Creatine Biosynthesis)
4-hydroxyproline Degradation I
L-serine Degradation
Intrinsic Prothrombin Activation Pathway
Branched-chain α-keto acid Dehydrogenase Complex
D-myo-inositol (1,4,5,6)-Tetrakisphosphate Biosynthesis
D-myo-inositol (3,4,5,6)-tetrakisphosphate Biosynthesis
2-ketoglutarate Dehydrogenase Complex
2-oxobutanoate Degradation I

Supplementary File 4. IPA analysis for pathways enriched at 24 wks (Top 10 pathways). L and H represent low and high diet respectively. The colour of each pathway (bar) indicates its direction of change; blue (positive z score)- up-regulated, orange (negative z score)- down-regulated, grey- no activity pattern available or white z score=0 (white). Different pathways are marked on the X-axis and -log(p value) marked on the Y-axis. Significant differences were measured in 2 ways: 1) Ratio (orange squares)- number of molecules from the data set that map to the pathway divided by the total number of molecules that map to the canonical pathway is displayed. 2) Fisher's exact test was used to calculate a p-value determining the probability that the association between the genes in the dataset and the canonical pathway is explained by chance alone (P< 0.05). The pathways were generated through the use of "core analysis" in IPA (QIAGEN Inc., https://www.qiagenbioinformatics.com/products/ingenuity-pathway-analysis) [1].

Reference

1. Krämer, A., Green, J., Pollard, J. Jr., Tugendreich, S. Causal analysis approaches in Ingenuity Pathway Analysis. *Bioinformatics* **30,** 523-530 (2014).
